# Characteristics Associated with Self-Reported Exercise among US Adults Age ≥50 Years with Self-Reported Pain in the Past Four Weeks Who Used an Opioid

**DOI:** 10.3390/healthcare11081129

**Published:** 2023-04-14

**Authors:** David R. Axon, Miles D. Quigg

**Affiliations:** 1 Department of Pharmacy Practice & Science, R. Ken Coit College of Pharmacy, The University of Arizona, 1295 N. Martin Ave., Tucson, AZ 85721, USA; milesquigg@arizona.edu; 2 Center for Health Outcomes and PharmacoEconomic Research (HOPE Center), R. Ken Coit College of Pharmacy, The University of Arizona, 1295 N. Martin Ave., Tucson, AZ 85721, USA

**Keywords:** physical activity, narcotic use, older people, Medical Expenditure Panel Survey

## Abstract

The number of older adults in the United States is growing, alongside the number of older adults experiencing some sort of pain and using opioids. Exercise is an important pain management and pain prevention strategy. However, little is known about the factors associated with exercise among United States adults ≥50 years old with pain who use opioids. This retrospective cross-sectional database study aimed to identify characteristics associated with self-reported frequent exercise (moderate- to vigorous-intensity exercise ≥30 min five times a week) in United States adults ≥50 years old with pain in the past four weeks who had also used an opioid. The study used 2020 Medical Expenditure Panel Survey data and logistic regression models. Analyses maintained the structure of the complex survey data and were weighted to obtain nationally representative estimates. Significantly associated variables with frequent exercise in the fully adjusted analysis included being aged 60–69 (versus ≥80 years, adjusted odds ratio [AOR] = 2.3, 95% confidence interval [CI] = [1.1–5.1]), having excellent/very good/good (versus fair/poor) self-perceived health (AOR = 2.4, 95% CI = [1.3–4.2]), normal/underweight (versus obese (AOR = 2.1, 95% CI = [1.1–3.9])), overweight (versus obese (AOR = 1.7, 95% CI = [1.0–2.9])), and having little (versus extreme) pain (AOR = 2.4, 95% CI = [1.0–5.7]). A secondary finding was that 35.7% considered themselves frequent exercisers, while the remaining 64.3% did not consider themselves frequent exercisers. In future, these findings can be used to personalize pain management strategies and encourage greater levels of exercise among this population.

## 1. Introduction

According to the International Association for the Study of Pain, pain is defined as “an unpleasant sensory and emotional experience associated with, or resembling that associated with, actual or potential tissue damage” [1]. Pain can come in a multitude of different forms. Acute pain comes on suddenly, usually due to disease, injury, or inflammation, and can be diagnosed or treated accordingly [2]. Chronic pain is defined as pain that recurs or persists for three months or longer. However, there are seven categories of chronic pain, each with distinct characteristics [3]. Chronic pain can be lifelong and cause severe detriments to quality of life and individual functioning [2].

In 2010, the estimated overall cost of pain in the United States (US) ranged from USD 560 to USD 635 billion, and the value of lost productivity was estimated to range from USD 299 to USD 335 billion [4]. The overall cost of pain exceeded the cost of some of the most common chronic conditions such as heart disease (USD 309 billion), cancer (USD 243 billion), and diabetes (USD 188 billion). Regardless of acute or chronic status, pain is associated with a negative impact on several outcomes, including daily activities, leisure activities, work activities, and relationships [5]. This often leads individuals with pain to pay for services such as housekeeping, childcare, and gardening that they would otherwise tend to themselves [6].

Despite the myriad of problems caused by pain, it is one of the most common conditions affecting US adults today. One study demonstrated that, in 2019, 20.5% of US adults had some degree of chronic pain, with 10% suffering from high-impact chronic pain that presented limitations to daily functioning [7]. These findings are consistent with a previous paper published in 2016 that calculated 20.4% of US adults had some degree of chronic pain and 8% had limitations due to pain [8].

Given the prevalence of pain, the management of pain is also important. There are a variety of different pain management strategies available [5,9]. One common pharmacological pain management strategy is opioids. Opioids have been used throughout history for pain management, yet, in the US, their use has increased over the past couple decades and led to the “opioid crisis”. Thus, balancing the benefits and risks of opioid use remains a challenge for healthcare professionals and policy makers [10,11]. According to chronic pain management guidelines, opioids are not typically regarded as a first line treatment [12]. However, in the National Comprehensive Cancer Network (NCCN) guidelines, opioids are preferred, in cancer, over non-steroidal anti-inflammatory drugs (NSAIDs) and acetaminophen, primarily due to dose related toxicities [13]. In addition, NSAIDs, topical agents, anti-depressants, and anticonvulsants typically have poor to modest efficacy [14]. In a 2018 meta-analysis, it was shown that individuals with chronic pain who used opioids had improved role functioning, social functioning, and sleep quality compared to placebo [15]. Yet opioids are far from ideal, as they are associated with increased mortality. In 2008, opioids were involved in 14,800 deaths in the US, which accounted for 73.8% of all prescription drug overdose deaths [16]. Since then, opioid related deaths have increased, with deaths due to synthetic opioids rising to 9.9 deaths per 100,000 people in 2018 [17].

Outside of pharmacological pain management strategies, exercise is a common non-pharmacological pain management strategy [9]. Exercise is an important pain management (and pain prevention) strategy because it reduces sedentariness, which is a key risk factor for developing chronic pain, in addition to improving health generally [18,19,20]. An overview of systematic reviews published in 2012 showed that frequent exercise improved overall pain in a wide variety of situations and established the necessity of exercise as a mainstay non-pharmacological pain management strategy [21]. In addition to improvements in pain, frequent exercise is associated with improvements in physical function, sleep, fatigue, anxiety, depression, and inflammation [14].

One of the largest growing demographics in the US is older adults. Using census data from 2012, it was projected that, in 2014, there would be almost 110 million adults aged 50 and over in the US [22]. In 2016, the Centers for Disease Control and Prevention (CDC) found that 27.5% of adults aged 50 and over (about 31 million people) reported no physical activity outside of work, and the percentage increased as age increased [23].

There is a lack of information about the factors associated with exercise among US adults aged ≥50 years old with pain who use an opioid. This is important to investigate because pain is particularly common among older US adults, with one study reporting that 38.5% of adults over the age of 65 and 47.7% of adults over the age of 85 had chronic pain [24]. Furthermore, opioids present an increased risk of adverse events, particularly when used in older adults [25], a population that is rapidly increasing [18]. However, it has been demonstrated that exercise can significantly reduce chronic pain while promoting good physical health at the same time, especially among older adults [26]. Therefore, the objective of this study was to assess the association between self-reported exercise and the characteristics of US adults age ≥50 years with self-reported pain in the past four weeks who used an opioid.

## 2. Methods

### 2.1. Study Design

The study employed a retrospective, cross-sectional design that used the 2020 Medical Expenditure Panel Survey (MEPS) data.

### 2.2. Medical Expenditure Panel Survey

MEPS provides a very comprehensive dataset and is conducted by the Agency for Healthcare Research and Quality (AHRQ) over the course of two calendar years and multiple rounds of interviews [27,28]. As a result, MEPS data can produce nationally representative estimates of trends in the non-institutionalized US population. MEPS has multiple components, including the MEPS household component (MEPS-HC), MEPS insurance component (MEPS-IC), and MEPS medical provider component (MEPS-MPC). MEPS-HC has detailed data regarding demographic information about every person in the households surveyed, such as education status, employment status, income level, health status, and many more [27,28]. In the MEPS survey, participants were asked specific questions that provided the necessary detail to answer each question. MEPS-IC is a survey given to employers in the US to collect data on the number and type of insurance plans offered, the benefits associated with the plans, the annual premiums, the contributions made by employers and employees, the eligibility requirements, and employer characteristics [22]. MEPS-MPC collects information from medical providers who provide care to MEPS-HC respondents, including physicians, pharmacies, hospitals, and home health agencies [22]. This study employed two data files: The MEPS 2020 full-year consolidated data file [28] and the MEPS 2020 prescribed medicines file [29]. MEPS is reviewed by an institutional review board annually, and all participants provide oral consent to participate before completing the survey [27]. This study was approved by The University of Arizona Institutional Review Board (Study #00002144, 18 November 2022).

### 2.3. Study Eligibility

All 27,805 individuals in the dataset were screened against the study eligibility criteria. Participants from the MEPS 2020 dataset were eligible for study inclusion if they were alive for the full calendar year, were 50 years old or older, reported having pain in the past four weeks, and had an opioid medication in the prescribed medicines file. To characterize pain, participants were asked “During the past four weeks, how much did pain interfere with your normal work (including both work outside the home and housework)?” [27]. Responses of “a little bit”, “moderately”, “quite a bit,” or “extremely” indicated the person had pain. To determine use of an opioid medication, Multum Lexicon therapeutic class codes of “60” (narcotic analgesics) or “191” (narcotic analgesic combinations) in the 2020 prescribed medicines file were used [27]. Adults aged 50 and older were chosen because the CDC recently recommended adults aged ≥50 need more exercise [30].

### 2.4. Variables

Using Andersen’s Behavioral Model, three groups of independent variables were assessed in this study [31].

Predisposing factors included: age in years (50–59, 60–69, 70–79, ≥80); gender (male, female); race (white, other); and ethnicity (Hispanic, non-Hispanic) [28].

Enabling factors included: marital status (married, other marital status); education level completed (high school or less, more than high school education); employment status (employed, unemployed); and income level (used as an indicator of poverty level; low income (<200% federal poverty level), middle income (≥200% federal poverty level), high income (≥400% federal poverty level)) [28].

Need factors included: the number of chronic conditions (from the MEPS list of highly prevalent conditions: angina, arthritis, asthma, cancer, chronic bronchitis, coronary heart disease, diabetes, joint pain, emphysema, hypercholesterolemia, hypertension, myocardial infarction, other unspecified heart disease, and stroke; <2, 2, 3, 4, 5, ≥6); perceived physical health status (excellent/very good/good, fair/poor); perceived mental health status (excellent/very good/good, fair/poor); smoker status (yes, no); body mass index (BMI) classification (normal/underweight, overweight, obese); limitations to activities of daily living (ADL; yes, no); limitations to instrumental activities of daily living (IADL; yes, no); and pain intensity (little, moderate, quite a bit, extreme). ADLs included help with bathing, dressing, eating, getting in or out of bed, mobility inside the home, or toileting. IADLs included using the telephone, paying bills, taking medicines, preparing light meals, doing laundry, or going shopping [28].

The outcome variable in this study was exercise status. Exercise was analyzed dichotomously (yes, no) depending on participants self-reported answer to the question, “Do you participate in at least 30 min of moderate- to vigorous-intensity physical activity at least five times per week?” [27,28].

### 2.5. Statistical Analysis

**Figure 1:**
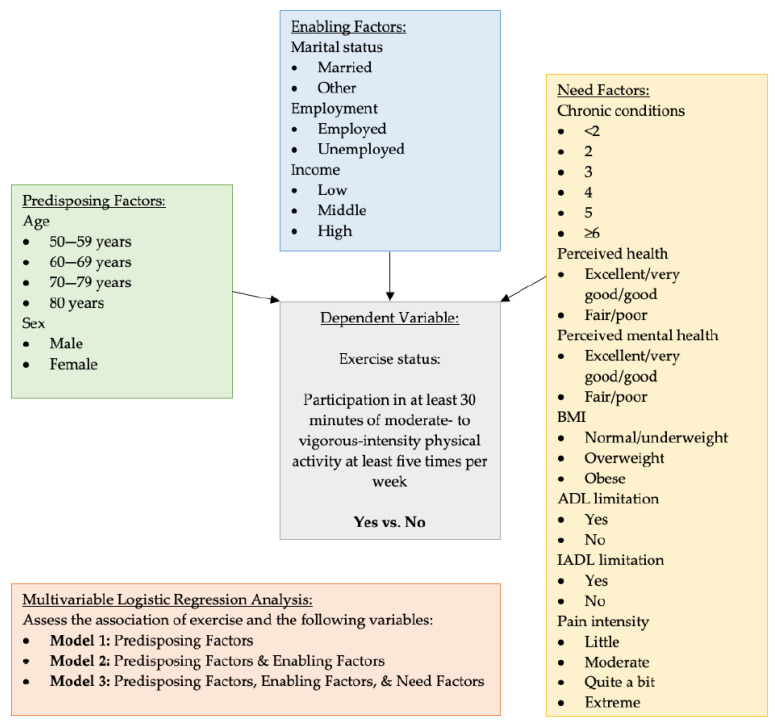
Overview of the statistical analysis approach. BMI = body mass index; ADL = activity of daily living; IADL = instrumental activity of daily living. Predisposing, enabling, and need factors developed by adapting Andersen’s Behavioral Model [31].

The MEPS cluster, strata, and weighting variables were used to account for the complexity of the MEPS design and to generate nationally representative population estimates. MEPS staff provide the cluster, strata, and weighting variables in the dataset for use by researchers in the analysis. The weighting is applied to all variables specified in the analysis. A detailed description of how the weighting variables were developed and how they should be applied by the researcher is provided in the MEPS documentation [27]. Nominal data were compared using chi-square tests. Multivariable logistic regression models were used to determine statistically significant associations between predisposing, enabling, and need factors with exercise status. Only variables that were shown to be significant in the univariate analysis were included in the multivariable logistic regression models. Logistic regression model 1 included predisposing factors (age, sex). Logistic regression model 2 included predisposing factors and enabling factors (age, sex, marital status, employment, income). Logistic regression model 3 included predisposing factors, enabling factors, and need factors (age, sex, marital status, employment, income, chronic conditions, perceived health, perceived mental health, BMI, ADL limitation, IADL limitation, pain intensity). The participants who reported that they exercised regularly were modeled, while those who reported that they did not exercise regularly were used as the referent group. Adjusted odds ratios and 95% confidence intervals (CI) were calculated. Standard errors were calculated using the Taylor-series linearization approach. An alpha level of 0.05 was set a priori. Missing data for some variables are inputted by MEPS staff when possible following MEPS coding conventions, which can include supplemental data from the MEPS insurance component, medical provider component, or earlier interview panels [28]. Any missing data remaining were excluded from analyses. The analyses were conducted using SAS on demand for academics (SAS Institute Inc., Cary, NC, USA) and PROC SURVEY commands to apply the cluster and strata variables. An overview of the analytical approach is provided in Figure 1.

## 3. Results

From a total of 27,805 people in the 2020 MEPS dataset, 843 were eligible for inclusion in this study (exercise status: yes = 288, no = 555). The sample was weighted to produce a weighted population estimate of 10,596,166 US adults aged ≥50 years old with self-reported pain in the past four weeks who used an opioid, of which 35.7% [95% confidence interval (CI) = 31.6%–39.9%] reported themselves as frequent exercisers (moderate- to vigorous-intensity exercise for at least 30 min five times a week).

Table 1 summarizes the characteristics of the study participants, differentiated by self-reported exercise status. Most respondents were female, white, non-Hispanic, had higher than high school education, were unemployed, perceived their physical and mental health as good, were nonsmokers, and reported no issues with ADL or IADL. Other collected demographic variables were age, marital status, income, number of chronic health conditions, BMI, and severity of the described pain, but there was no clear majority in these groups. Between the exercise and non-exercise groups, there was no significant difference (*p* ≥ 0.05) in race, ethnicity, education status, and smoking status. There were significant differences between groups for all other variables (age, sex, marital status, employment status, income, chronic conditions, physical health status, mental health status, BMI, ADL limitations, IADL limitations, and pain, *p* < 0.05).

Table 2 demonstrates the results of the logistic regression analyses. Among the predisposing factors, those aged 60–69 years had approximately two times greater odds of reporting doing frequent exercise compared to those aged ≥80 years in all three models. Males had 1.5 times greater odds of reporting doing frequent exercise compared to females in the initial model (model 1), but this association was no longer significant is subsequent models (models 2 and 3). None of the enabling factors were significantly associated with exercise in the fully adjusted analysis, although those who were employed had greater odds of reporting doing frequent exercise compared to those who were unemployed in model 2. However, this effect was not sustained in the fully adjusted analysis. Among the need factors, those who reported they were in excellent/very good/good health had 2.4 times greater odds of reporting doing frequent exercise compared to those who reported they were in fair/poor health in the fully adjusted analysis. Likewise, those who reported they were normal/underweight and overweight had 2.1 times greater odds and 1.7 times greater odds, respectively, of reporting doing frequent exercise compared to those who reported they were obese in the fully adjusted analysis. Furthermore, those who reported they had little pain had 2.4 times greater odds of reporting doing frequent exercise compared to those who reported they had extreme pain in the fully adjusted analysis.

## 4. Discussion

This study showed, in the fully adjusted analysis, that US adults aged 60–69 who had pain and used an opioid had greater odds of reporting doing frequent exercise than those aged ≥80 years. This finding is supported by a report about physical activity that indicated older adults perform less physical activity as they age [23]. Likewise, a Canadian retrospective data review study showed individuals were less likely to exercise if they were older [32]. However, it is interesting that the other age groups in this study were not associated with frequent exercise. It may be that some of the people in the 60–69-year-old group are retired or semi-retired, still in good enough health, and have the time for frequent exercise, whereas those in the 50–59-year-old group may be too busy with work to exercise frequently, and those in the over 70 year old group may not have good enough health to exercise frequently.

This study also showed, in the fully adjusted analysis, that those with excellent/very good/good perceived health had greater odds of reporting doing frequent exercise than those with fair/poor perceived health. Again, this finding correlates with the findings reported by a study of US adults aged ≥50 years with pain in the 2017 MEPS data [33]. The association between health status and exercise status is perhaps unsurprising given that one of the benefits of frequent exercise is improved health status [34]. However, these findings should be interpreted with caution. A recent poll found that 79% of US adults polled reported themselves as being in good or excellent physical health [35], while a European study using 2013 data from 17 different countries showed that individuals tended to overestimate or underestimate their physical health status in different countries [36]. Research to verify the actual health status of US adults aged ≥50 years with pain who used an opioid is therefore needed to add extra validity to these findings.

In addition, this study showed, in the fully adjusted analysis, that those who were normal/underweight or those who were overweight had greater odds of reporting doing frequent exercise than those who were obese. Previous work has shown greater levels of inactivity among those who are obese [23], and that exercising for more than 150 min per week is associated with a lower risk of obesity [37]. Given our study population of US adults aged ≥50 years with pain who used opioids, it may be that those who are obese are in considerable levels of pain and are therefore unable to exercise frequently. Since obesity is a modifiable risk factor for health, interventions could be targeted at obese people to assess the root causes of their weight and help improve their health.

Furthermore, this study showed, in fully adjusted analysis, that US adults aged ≥50 years with pain who used an opioid and reported little pain intensity had greater odds of reporting doing frequent exercise than those who reported extreme pain intensity. The association between pain and exercise is a complex one, with several examples reported in the recent literature [38,39,40,41]. On one hand, greater levels of pain may prevent older adults from being able to exercise frequently. For instance, previous work has shown greater levels of pain with increased levels of exercise among a variety of populations with pain, including women with fibromyalgia [38] and among patients with knee pain [39]. On the other hand, exercise may help alleviate pain. For instance, those who exercised at least three times per week were more likely to experience positive pain outcomes after exercise [40], while another study from Germany during the COVID-19 pandemic showed that exercising more than four hours per week reduced the likelihood of experiencing severe pain [41].

In this study, none of the remaining variables were associated with frequent exercise. Previous research has found that some of these variables are negatively associated with frequent exercise status in other populations. For example, a Canadian retrospective data review showed that, despite physical exercise having a positive effect on depression and anxiety symptoms, 50% of respondents did not exercise regularly [32]. The same study showed individuals were less likely to exercise if they were less educated or had lower income [32]. Another study showed that persistent smokers exercised less and had decreasing exercise habits compared to individuals who never smoked [42].

Also, there were no associations between other variables (e.g., gender, ethnicity, employment, chronic conditions) that have previously been identified as being associated with exercise status among US adults aged ≥50 years with pain [33]. The lack of these associations in the current study are most likely due to the very specific eligibility criteria used, i.e., US adults aged ≥50 years old with pain who used an opioid. Future research is therefore warranted to establish an association between various personal characteristics and opioid use and to further investigate the effect of opioid use on exercise status.

A secondary finding from this study was that approximately 36% of US adults aged ≥50 years with self-reported pain who had used an opioid reported doing 30 or more minutes of moderate–high intensity exercise at least five times per week. From a healthcare perspective, it is concerning that only just over one-third of US adults aged ≥50 years old reported doing frequent exercise. However, the individuals in this study did report having pain and using an opioid; thus, it is possible that their pain prevented them from participating in regular exercise. A previous study showed that the proportion of adults aged >65 years reporting frequent exercise ranged from approximately 36% to 44% [43], which is similar to our finding. However, another MEPS analysis of a similar population without opioid use showed that the proportion of US adults ≥50 years reporting frequent exercise was higher than in the current study, at approximately 42% [33]. This finding may be explained by the US adults aged ≥50 years with pain in the current study using opioids, which could indicate greater severity of pain, and thus, perhaps, less ability to exercise frequently. The findings of the current study, supported by other recent studies in similar populations, suggest the need to increase frequent exercise among US adults aged ≥50 years old with pain who used an opioid.

There were some limitations of this study. First, this retrospective study was not able to establish a causal relationship between variables; rather, only a statistical association can be shown. Second, the self-reported data provides an opportunity for recall bias. Third, exercise and pain were defined based on the items available in MEPS. The exercise item only asked about frequency and intensity of exercise and was not able to distinguish between types of exercise. Similarly, the pain item was not able to distinguish between various pain characteristics such as type of pain, duration of pain, locality of pain, etc. Fourth, opioid use was defined based on whether the individual reported using an opioid in the calendar year, with no distinction between type of opioid or acute use (e.g., opioid use for a few days following surgery) versus chronic use (e.g., daily opioid use for chronic pain). However, the strengths of the study include the use of a large nationally representative sample of the US population, which means that findings can be extrapolated to the wider population, and the use of the most recently available MEPS 2020 data.

## 5. Conclusions

Among US adults over the age of 50 who reported pain in the past four weeks and who also reported using an opioid in the calendar year, age 60–69 versus age ≥80 years, excellent/very good/good versus fair/poor perceived health status, normal/underweight or overweight versus obese, and little versus extreme pain had greater odds of reporting doing ≥30 min of moderate-vigorous intensity exercise ≥5 times per week. In addition, approximately 36% of US adults aged ≥50 with pain who used an opioid reported doing ≥30 min of moderate-vigorous intensity exercise ≥5 times per week. Future research to establish the relationship between these variables and opioid use may be helpful to further explain these findings, while the relatively low levels of frequent exercise reported in this study indicate a need to encourage greater levels of exercise among this population.

## Figures and Tables

**Table 1:** Characteristics of United States adults aged ≥50 years with self-reported pain in the past four weeks who used opioids stratified by self-reported exercise (frequent exercise versus no frequent exercise) in the 2020 Medical Expenditure Panel Survey (Weighted N = 10,596,166).

Variables		Frequent Exercise Sample Size (N = 288)	Frequent Exercise (Weighted N = 3,783,303)	No Frequent Exercise Sample Size (N = 555)	No Frequent Exercise (Weighted N = 6,812,863)	*p*
			Weighted % [95% CI]		Weighted % [95% CI]	
Age	50–59 years	70	30.2 [22.9–37.4]	161	31.1 [26.1–36.1]	0.044
	60–69 years	115	37.8 [30.4–45.2]	179	29.7 [25.0–34.4]	
	70–79 years	78	24.6 [18.3–30.8]	138	24.6 [19.8–29.5]	
	≥80 years	25	7.5 [4.2–10.8]	77	14.6 [10.9–18.4]	
Sex	Male	129	46.6 [40.2–53.0]	175	35.5 [30.1–41.0]	0.015
	Female	159	53.4 [47.0–59.8]	380	64.5 [59.0–69.9]	
Race	White	235	84.9 [79.6–90.1]	421	80.8 [77.2–84.4]	0.213
	Other	53	15.1 [9.9–20.4]	134	19.2 [15.6–22.8]	
Ethnicity	Hispanic	22	5.7 [2.6–8.8]	50	6.3 [3.9–8.8]	0.747
	Non-Hispanic	266	94.3 [91.2–97.4]	505	93.7 [91.2–96.1]	
Marital status	Married	137	56.8 [49.4–64.2]	208	46.4 [41.7–51.1]	0.023
	Other	151	43.2 [35.8–50.6]	347	53.6 [48.9–58.3]	
Education	High school or less	146	45.6 [38.8–52.3]	295	49.8 [44.5–55.1]	0.362
	More than high school	141	54.4 [47.7–61.2]	256	50.2 [44.9–55.5]	
Employment	Employed	93	38.2 [31.2–45.2]	110	22.7 [18.4–27.1]	<0.001
	Unemployed	195	61.8 [54.8–68.8]	445	77.3 [72.9–81.6]	
Income	Low	117	32.6 [25.8–39.5]	270	39.8 [34.8–44.9]	0.005
	Middle	65	22.4 [16.7–28.2]	152	29.4 [24.5–34.2]	
	High	106	44.9 [37.7–52.2]	133	30.8 [25.1–36.6]	
Chronic conditions	<2	38	17.1 [11.8–22.5]	41	7.6 [4.9–10.2]	0.001
	2	49	16.5 [11.7–21.3]	73	14.5 [10.7–18.2]	
	3	67	22.4 [16.0–28.8]	108	19.9 [15.8–24.1]	
	4	47	13.7 [9.1–18.3]	121	20.3 [16.3–24.4]	
	5	39	15.9 [10.5–21.4]	85	16.0 [12.1–19.9]	
	≥6	48	14.3 [9.7–18.9]	127	21.7 [17.7–25.7]	
Perceived health	Excellent/very good/good	215	77.4 [71.1–83.6]	285	50.8 [46.0–55.5]	<0.001
	Fair/poor	73	22.6 [16.4–28.9]	270	49.2 [44.5–54.0]	
Perceived mental health	Excellent/very good/good	251	89.3 [85.3–93.4]	416	74.8 [70.3–79.4]	<0.001
	Fair/poor	37	10.7 [6.6–14.7]	139	25.2 [20.6–29.7]	
Smoker	Yes	39	13.7 [8.3–19.1]	103	16.2 [13.0–19.4]	0.465
	No	249	86.3 [80.9–91.7]	451	83.8 [80.6–87.0]	
BMI	Normal/underweight	75	28.4 [20.9–35.9]	111	21.6 [17.5–25.6]	0.003
	Overweight	98	35.3 [28.1–42.6]	137	27.6 [22.8–32.5]	
	Obese	107	36.3 [30.0–42.6]	279	50.8 [45.7–56.0]	
ADL limitation	Yes	8	2.7 [0.7–4.6]	33	10.3 [6.7–13.8]	<0.001
	No	190	97.3 [95.4–99.3]	318	89.7 [86.2–93.3]	
IADL limitation	Yes	14	7.8 [3.2–12.3]	60	16.9 [12.1–21.7]	0.023
	No	184	92.2 [87.7–96.8]	290	83.1 [78.3–87.9]	
Pain intensity	Little	105	35.0 [28.6–41.3]	122	23.1 [19.0–27.3]	<0.001
	Moderate	79	31.3 [23.3–39.3]	129	24.6 [19.9–29.3]	
	Quite a bit	77	24.9 [18.7–31.0]	180	30.6 [26.4–34.7]	
	Extreme	27	8.9 [4.5–13.3]	124	21.7 [17.6–25.7]	

Analysis based on an unweighted sample *n* = 843 (frequent exercise *n* = 288; no frequent exercise *n* = 555) of United States adults alive during the calendar year 2020, age ≥ 50 years, with self-reported pain in the past four weeks who used at least one opioid. Sample sizes do not always total 100% due to missing data. Statistically significant differences between groups based on chi-square tests. BMI = body mass index. ADL = activities of daily living. IADL = instrumental activities of daily living. CI = confidence interval.

**Table 2:** Characteristics associated with frequent exercise (versus no frequent exercise) among United States adults aged ≥50 years with self-reported pain in the past four weeks who used opioids in the 2020 Medical Expenditure Panel Survey.

Factors	Model 1 AOR (95% CI)	Model 2 AOR (95% CI)	Model 3 AOR (95% CI)
Age 50–59 years	1.9 [1.0–3.6]	1.4 [0.7–2.9]	1.6 [0.7–3.9]
Age 60–69 years	**2.4 [1.3–4.4]**	**2.0 [1.1–3.8]**	**2.3 [1.1–5.1]**
Age 70–79 years	1.9 [1.0–3.7]	1.7 [0.8–3.3]	1.4 [0.6–3.2]
Age ≥ 80 years	Ref	Ref	Ref
Male sex	**1.5 [1.1–2.2]**	1.5 [1.0–2.2]	1.5 [0.9–2.5]
Female sex	Ref	Ref	Ref
Married		1.2 [0.8–1.8]	1.2 [0.7–2.0]
Other marital status		Ref	Ref
Employed		**2.0 [1.2–3.1]**	1.5 [0.8–2.9]
Unemployed		Ref	Ref
Low income		0.8 [0.5–1.3]	1.1 [0.6–2.0]
Middle income		0.6 [0.4–1.0]	0.7 [0.4–1.3]
High income		Ref	Ref
<2 chronic conditions			1.6 [0.6–4.3]
2 chronic conditions			1.1 [0.5–2.5]
3 chronic conditions			1.1 [0.5–2.3]
4 chronic conditions			0.7 [0.3–1.5]
5 chronic conditions			1.4 [0.6–3.1]
≥6 chronic conditions			Ref
Excellent/very good/good perceived health			**2.4 [1.3–4.2]**
Fair/poor perceived health			Ref
Excellent/very good/good perceived mental health			1.0 [0.5–2.1]
Fair/poor perceived mental health			Ref
Normal/underweight			**2.1 [1.1–3.9]**
Overweight			**1.7 [1.0–2.9]**
Obese			Ref
ADL limitation			0.5 [0.1–1.4]
No ADL limitation			Ref
IADL limitation			1.1 [0.4–2.7]
No IADL limitation			Ref
Little pain intensity			**2.4 [1.0–5.7]**
Moderate pain intensity			1.8 [0.8–4.3]
Quite a bit pain intensity			1.5 [0.6–3.3]
Extreme pain intensity			Ref

Analysis based on an unweighted sample *n* = 843 (frequent exercise *n* = 288; no frequent exercise *n* = 555) of United States adults alive during the calendar year 2020, age ≥ 50 years, with self-reported pain in the past four weeks who used at least one opioid. The reference group in the binomial logistic regression models was no frequent exercise. Model 1 had a c-statistic of 0.6. Model 2 had a c-statistic of 0.6. Model 3 had a c-statistic of 0.7. Bold indicates the characteristic has a significant association with frequent exercise. AOR = adjusted odds ratio. CI = confidence interval. Ref = Reference group. BMI = body mass index. ADL = activities of daily living. IADL = instrumental activities of daily living.

## Data Availability

Data are available from the corresponding author upon reasonable request.
